# State of the science on controversial topics: missing maxillary lateral incisors - a report of the Angle Society of Europe 2012 meeting

**DOI:** 10.1186/2196-1042-14-20

**Published:** 2013-07-26

**Authors:** Ama Johal, Christos Katsaros, Anne Marie Kuijpers-Jagtman, Angle Society of Europe membership

**Affiliations:** Department of Oral Growth and Development, Institute of Dentistry, Queen Mary College, Turner Street, London, E1 1BB UK; Department of Orthodontics and Dentofacial Orthopedics, Medical School, University of Bern, Freiburgstr. 7, Bern, CH-3010 Switzerland; Department of Orthodontics and Oral Biology, Cleft Palate Craniofacial Unit, Radboud University Nijmegen Medical Centre, 309 Dentistry, PO Box 9101, Nijmegen, 6500, HB The Netherlands; P.za della Mostra, 19, Trento, 38122 Italy

## Abstract

**Background:**

The optimal long-term management of the congenitally missing maxillary lateral incisor continues to cause controversy within the specialty. The Angle Society of Europe meeting 2012 dedicated a day to address some of the current controversies relating to the management of these missing lateral incisors.

**Findings:**

The format of the day consisted of morning presentations and afternoon breakout sessions to discuss a variety of questions related to the management of missing lateral incisors.

**Conclusions:**

The consensus viewpoint from this day was that the care of patients with congenitally missing lateral incisors is best achieved through a multi-disciplinary approach. The current evidence base is weak, and further well-designed, prospective trials are needed.

## Findings

### Introduction

The optimal long-term management of the congenitally missing maxillary lateral incisor continues to cause controversy within the specialty. Opinions remain divided, as evidenced by the ‘point/counterpoint’ discussion recently published in the *American Journal of Orthodontics and Dentofacial Orthopedics* as to whether to open or close the resultant space with either a restorative replacement or canine substitution, respectively [1, 2]. In light of this, the Angle Society of Europe (ASE) meeting 2012 dedicated a day to address some of the current controversies relating to the management of these missing lateral incisors.

### Methods

The format of the day was that the *morning session* consisted of the following seven presentations by leading clinicians from within the society, followed by a 10-min open discussion. Then a number of questions were posed for further discussion in the afternoon breakout sessions in relation to the following topics:

*Thor Henrikson*: ‘Agenesis of upper laterals, overview of treatment options’

Question 1. Is there enough scientific evidence to decide what to do?

*Marco Rosa*: ‘Space closure: treatment considerations’

Question 2. What are the priorities to be considered in the treatment plan: gummy smile? age of the patient? malocclusion? size of teeth? patient’s expectations?

Question 3. Both space closure and implant substitution are difficult, long and expensive treatment options. Do we have simple and cheap alternatives?

*Björn Zachrisson*: ‘Space closure: is this the preferred treatment option-1?’

Question 4. What to do when you do not have a good prosthodontist to work with on your case.

Question 5. What to do when you have a referral for implant, but you are in favour of space closure.

*Domingo Martin*: ‘Space opening: is this the preferred treatment option-2?’

Question 6. What are some important aspects to take into account before placing implants in the anterior region?

Question 7. What aspects are crucial for success in implant placement in the anterior region?

*Stavros Kiliaridis*: ‘Implant substitution: considerations on long-term results’

Question 8. Unilateral versus bilateral missing laterals: treatment aspects to take into consideration.

*Guiliano Maino*: ‘Missing laterals: periodontal considerations’

Question 9. How important are the patient’s biotype and height and thickness of the interdental papillae for your treatment plan strategy?

*Christos Katsaros*: ‘The patient’s perspective’

The *afternoon session* involved the ASE members forming into seven breakout groups and an appointed Chair, with each group being assigned to address one or more of the above questions and present back to the whole group their conclusions during the final session of the evening. The findings in relation to each question were addressed in turn by the group(s) to which they were assigned, and a short but very interactive whole-group discussion followed. Ama Johal summarised the activity of the day and drew the following conclusions in relation to each question as follows:

Question 1. Is there enough scientific evidence to decide what to do?

The current evidence base was judged to be relatively weak. The research available to date is both clinical and patient-based but is predominately retrospective in nature. In evaluating the evidence in relation to space closure, it would appear that this results in a more favourable periodontal status and reported patient satisfaction too [3–6].

In relation to opening space for absent upper lateral incisors, the option of a resin-retained cantilever bridge design shows good long-term survival rates, but there remains a potential for rotational relapse of the canine and subsequent poor aesthetics. With respect to a single implant-stabilised prosthetic replacement, there appears to be longitudinal data to demonstrate high levels of unpredictability, in terms of the risks of progressive infraocclusion and marginal bone loss, compromising aesthetics [7, 8]. In view of the weak evidence regarding treatment of missing upper laterals, prospective clinical trials are much needed to give further light and guidelines to clinicians.

Question 2. What are the priorities to be considered in the treatment plan: gummy smile? age of the patient? malocclusion? size of teeth? patient’s expectations?

This was considered a multi-factorial issue with a large number of variables. From the choices that were given in the original question, there was some consensus in the group that the priority was space closure where patients had a harmonious exposure of gingival tissues during normal function (gummy smile) [9].

With the technical improvements in orthodontics that we have today, it was felt that all spaces could be closed. Alternatively, the space could be located far posteriorly in the dental arch [2]. The determining factors which have to be taken into consideration to come to a final decision could be a combination of risk of damaging tissues, treatment time, costs, aesthetic compromises, stability and morbidity to name but a few.

Question 3. Both space closure and implant substitution are difficult, long and expensive treatment options. Do we have simple and cheap alternatives?

The following options were considered as being appropriate:

1. No treatment if the patient expressed no concerns and there was an acceptable dental appearance.
2. Minimal treatment in the form of interim composite resin restorations could be considered if there was mild anterior spacing of concern to the patient.
3. In the presence of a malocclusion, no simple and cheap alternative could be offered.

Question 4. What to do when you do not have a good prosthodontist to work with on your case.

Question 5. What to do when you have a referral for implant, but you are in favour of space closure.

In response to questions 4 and 5, the overwhelming duty of care must still be the patient, and a mutli-disciplinary approach to management was preferred to facilitate informed consent [1]. Patients and parents need to be made aware of the relative advantages and disadvantages of each approach and guided as to what the team feels is most appropriate to the particular patients’ needs. If these are at odds with the referring practitioner, then the team/orthodontist must be prepared to ‘lose’ the referring practitioner in the best interest of the patient. An attempt should be made to ‘educate’ the referring dentist. One method could include demonstrating to the dentist the ‘alternative’ options and their outcomes compared to the original request of the dentist.

Question 6. What are some important aspects to take into account before placing implants in the anterior region?

The following considerations were thought to be important:

1. Implants could be contraindicated in light of the patient’s medical history and/or sustained smoking habit.
2. A 3D evaluation of the available bone using CBCT (or equivalent) was crucial [10].
3. Await skeletal maturity [8].
4. Orthodontic treatment delayed as long as possible, taking account of the patient’s concerns.
5. Appropriate retention was important, particularly following placement of a resin-retained bridge if orthodontic treatment was undertaken ‘early’ [1].
6. The presence of a gummy smile and a long face were considered contraindications to implant placement [9].
7. The patient’s sporting activity should be taken account of in planning the type of replacement. The preference was again to consider implant replacement in the posterior regions of the dentition.
8. Consider the use of a minimal-length anterior implant to permit a ‘plan b’ option, such as corticotomy and distraction, should a significant degree of infraocclusion take place.

Question 7. What aspects are crucial for success in implant placement in the anterior region?

A multi-disciplinary approach was preferable to management, and within this, the following considerations should be made to facilitate optimal implant placement:

1. Orthodontic treatment should facilitate the correct inter-crown and root distance, symmetry and root parallelism [11].
2. Retention should be carefully planned, and bonded retainers considered [1].
3. In situations where more than one tooth were absent, it was considered preferable to avoid placing two implants adjacent to each other.
4. Orthodontic treatment delayed as long as possible, taking account the patient’s concerns [8].
5. The surgical technique is demanding and may require connective tissue and bone grafts for a successful outcome [10, 12].
6. For adults, it is preferable to retain the fixed appliances *in situ* during the implant placement process in order to achieve optimal finishing.
7. The mother’s concerns were considered very important in the overall decision making process.

Question 8. Unilateral versus bilateral missing laterals: treatment aspects to take into consideration.

Important considerations to take account of were maintenance of midline symmetry and the crown morphology of the contra-lateral incisor, e.g. peg-shaped/microdont. A number of options were proposed based on the presenting situation:

Option 1. Extract the contra-lateral incisor and undertake orthodontic space closure if the following conditions are present:

1. There was a peg-shaped lateral incisor. An alternative option in the presence of a ‘normal’ lateral incisor could be to extract the contra-lateral second premolar.
2. Crowding on the contra-lateral arch.
3. A class II malocclusion.
4. Deviation of the centre line towards the absent lateral incisor.

Option 2. A non-extraction approach could be considered to either ‘open’ space if the following conditions are present:

1. Class I buccal segment relationship bilaterally.
2. Inclination of the upper incisors is favourable to proclination.
3. Class III camouflage is appropriate.
4. Spaced upper arch.
5. Lack of alveolar bone.

A non-extraction approach could be considered to either ‘close’ space if the following conditions are present:

1. Class II buccal segment relationship on the side of the arch with the absent lateral incisor.
2. Class II canine relationship on the side of the arch with the absent lateral incisor.

Question 9. How important are the patient’s biotype and height and thickness of the interdental papillae for your treatment plan strategy?

The above factors were considered very important to the long-term success of any treatment plan and again reinforced the need for a multi-disciplinary approach as being essential [1].

### Conclusions

The consensus viewpoint from this day was that the care of patients with congenitally missing lateral incisors is best achieved through a multi-disciplinary approach. It was acknowledged that a number of treatment options are available to the patient, including canine substitution, tooth-supported restorations and implant-supported restorations. Whilst each offers a number of advantages and disadvantages, there remains a relatively weak evidence base for these decisions, and further well-designed, prospective trials are needed.
